# Optional Vaccines, Collective Risk

**DOI:** 10.7759/cureus.104135

**Published:** 2026-02-23

**Authors:** Esteban Zavaleta-Monestel, Jeaustin Mora-Jiménez, Kevin Cruz Mora, Sebastián Arguedas-Chacón

**Affiliations:** 1 Department of Pharmacy, Hospital Clinica Biblica, San José, CRI; 2 Department of Research, Hospital Clinica Biblica, San José, CRI

**Keywords:** preventive medicine, public health communication, shared clinical decision-making, vaccine recommendations, vaccine uptake

## Abstract

Vaccination effectiveness depends not only on biological efficacy but also on how recommendations are framed and communicated in clinical practice. When vaccines are presented as routine standard of care, uptake remains high and responsibility is shared across clinicians, institutions, and communities. In contrast, framing vaccine recommendations as discretionary through shared clinical decision-making may unintentionally generate uncertainty, increase decisional burden for families, and reduce vaccine uptake. In a globally connected information environment, such ambiguity can extend beyond national borders, influencing vaccine confidence in health systems with limited capacity to manage uncertainty. This editorial argues that the issue lies not in shared decision-making itself, but in how “optional” framing is interpreted by clinicians and the public. Maintaining clear, routine vaccine recommendations is essential to sustaining trust, supporting efficient preventive care, and protecting population health.

## Editorial

The effectiveness of vaccination as a public health intervention depends not only on biological efficacy but also on the strength of the societal and clinical expectation that immunisation is a routine, normative act. Vaccination works best when it is perceived as standard care rather than a discretionary choice. When vaccination is presented as the expected standard, uptake remains high and responsibility is distributed among families, clinicians, and institutions. Clarity in recommendation framing supports trust, efficiency, and population-level protection. In contrast, when recommendations are framed as discretionary, the resulting consequences extend beyond individual choice and increasingly affect populations across national borders [1].

Recently, certain regions of the United States have adopted shared clinical decision-making (SCDM) for selected vaccine recommendations. Although this approach is often motivated by ethical considerations rather than scientific uncertainty, its practical effects extend beyond the consultation room. In a globally connected information environment, ambiguity originating from influential institutions spreads rapidly, influencing perceptions of vaccine confidence in health systems that may lack the necessary infrastructure, time, or trust to manage such uncertainty. As a result, what is intended as ethical flexibility may be interpreted as clinical hesitation. This move away from routine recommendation framing constitutes a destabilising shift in the global health landscape at a time when clarity is critical to addressing increasing vaccine hesitancy [2].

The ethical rationale for shared decision-making is evident, as it respects patient autonomy and offers a framework for addressing complex clinical scenarios where risk varies by individual. The concern raised here is not ethical intent, but communicative impact. The challenge lies not in SCDM itself, but in how discretionary framing is interpreted in practice. When a vaccine recommendation is viewed as optional, many parents may infer that the benefits are limited, the risks are significant, or that postponement is a reasonable interim decision. These interpretations are predictable responses to uncertainty, rather than indications of negligence or irresponsibility.

In this context, SCDM can function as a de facto de-recommendation. When a vaccine moves from a routine recommendation to a discretionary one, the public does not perceive flexibility; it perceives doubt. Evidence from the United States illustrates this effect. After the pneumococcal conjugate vaccine (PCV13) for immunocompetent adults aged 65 years or older was reclassified to SCDM in 2019, uptake among eligible beneficiaries declined substantially, despite no change in safety data [3]. For clinicians, “optional” language often signals marginal benefit; for patients, it implies meaningful risk.

**Figure 1:**
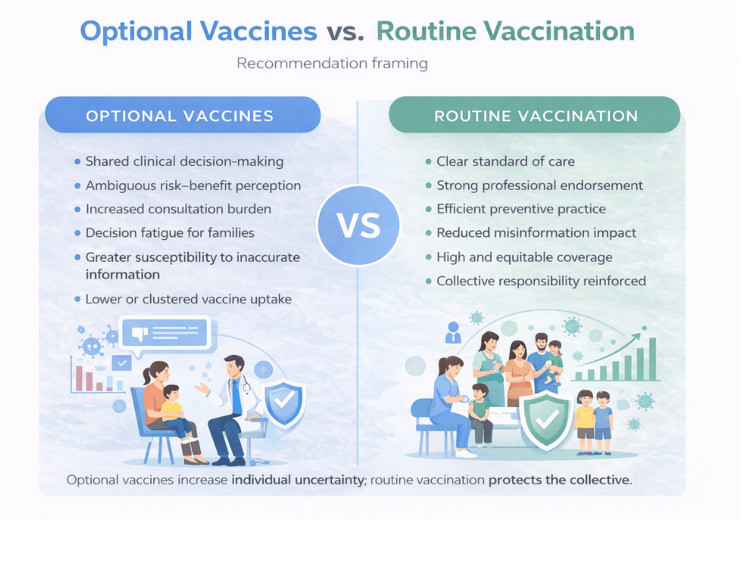
Optional Versus Routine Vaccine Recommendations: Individual Uncertainty and Collective Risk Source: authors’ own creation. This figure is an original conceptual illustration developed for this manuscript and based on concepts described in references [4] and [5]. The figure was created using Canva (Canva Pro, web-based graphic design platform, Sydney, Australia).

Figure 1 summarises the contrasting implications of discretionary versus routine vaccine recommendations. Optional framing increases individual uncertainty, decision burden, and susceptibility to inaccurate information, whereas routine recommendations reinforce clinical clarity, professional endorsement, and collective protection. This contrast illustrates how recommendation language alone can shape both individual behaviour and population outcomes [4].

For practising physicians, this shift changes a proactive endorsement into a prolonged ethical discussion. In the United Kingdom, where General Medical Council guidance on decision-making and consent emphasises shared responsibility, clinicians may become neutral conveyors of information rather than advocates for preventive care [5]. Given record-high workload pressures and the constraints of standard 10-minute consultations, this transformation is not neutral; it imposes additional and largely unsupported demands on clinical time and emotional resources. General practitioners are increasingly required to address complex vaccine misconceptions, often based on distorted risk perceptions or philosophical objections, without the support of unequivocal national recommendations [4].

This approach also imposes a considerable epistemic burden on families. Parents are implicitly required to evaluate rare adverse events against abstract, population-level benefits such as herd immunity. For many families, this represents an unrealistic expectation. In an environment saturated with information, this dynamic creates gaps that are easily exploited by misinformation. By shifting responsibility from systems to individuals, the healthcare system risks inducing choice fatigue and moral anxiety among those it aims to protect. Over time, shifting responsibility from institutions to individual consultations may erode, rather than strengthen, public trust [4].

Public health does not stop at national borders. Decisions made by high-salience bodies such as the US Centers for Disease Control and Prevention or the UK’s Joint Committee on Vaccination and Immunisation are global signals. Their guidance is amplified internationally through professional networks and media coverage, often detached from its original epidemiological context [6]. In many low- and middle-income countries, where health systems are overstretched and trust reserves are limited, the perception that a vaccine is “optional” can have disproportionate consequences. These systems rely on clear, routine norms to sustain the high coverage required for population protection.

Recent data indicate increasing vulnerability. In England, disparities in childhood vaccination uptake have widened, and coverage for several routine vaccines has fallen below target levels [7]. Measles, a sensitive indicator of immunisation system performance, continues to re-emerge internationally in areas with delayed or incomplete vaccination, illustrating how localised immunity gaps can quickly escalate into broader public health threats [8].

Behavioural science consistently demonstrates that the strength of recommendations serves as a default. Strong clinician endorsement and routine framing increase vaccine uptake, whereas discretionary language predictably reduces it, especially in contexts of uncertainty or misinformation. Vaccine recommendations thus extend beyond guiding individual decisions; they actively shape social norms. When vaccination is routine, refusal requires justification and protection is recognised as a collective responsibility. When vaccination is optional, deferral becomes socially acceptable and system failures often remain unnoticed until outbreaks arise [9].

None of this argues against SCDM where it is ethically and clinically justified, particularly in situations where risk-benefit profiles vary substantially across subgroups, where evidence is context-dependent, or where individual medical history meaningfully alters expected benefit. However, institutions whose recommendations carry international influence bear responsibility for how those recommendations are communicated. This includes clarity about evidentiary confidence, transparency about contextual assumptions, and safeguards against misinterpretation. Practical steps include maintaining routine recommendation framing as the default, reserving SCDM for vaccines with genuinely narrow clinical indications, providing standardised decision aids where discretion is required, and monitoring the equity and clustering effects of recommendation changes.

Vaccination constitutes a shared responsibility rather than a consumer choice. Maintaining clear, routine recommendations is not paternalistic; it is protective. Maintaining normative clarity is not paternalistic; it is a fundamental obligation to global public health. When clarity is lost, uncertainty is transmitted internationally, and its consequences are most acutely felt by those least equipped to address them.
